# Co-occurring intellectual disability and substance use disorders

**DOI:** 10.3934/publichealth.2021037

**Published:** 2021-06-17

**Authors:** Nita V Bhatt, Julie P Gentile

**Affiliations:** 1 Associate Director of Medical Student Education Psychiatry Wright State University Boonshoft School of Medicine Dayton, Ohio, USA; 2 Chair Department of Psychiatry Wright State University Boonshoft School of Medicine Dayton, Ohio, USA

**Keywords:** intellectual disability, developmental disability, substance use disorders, addiction

## Abstract

Individuals with intellectual disabilities (ID) are an expanding population that confronts multiple disadvantages from social and environmental determinants of health. Deinstitutionalization and community integration have improved the lives of individuals with ID in many ways. However, deinstitutionalization may increase their access to alcohol and drugs and the potential for developing Substance Abuse Disorders (SUD). It is estimated that 7–8 million people in the United States with an intellectual disability (ID) suffer disproportionately from substance use problems [1]. There is a lack of empirical evidence to inform prevention and treatment efforts in this population and more research needs to be done in order to address these issues.

## 1. Introduction

Individuals with ID often have under-diagnosed or under-treated medical conditions [1]. Although the prevalence of alcohol and illicit drug use is lower in this population, the risk of developing use disorders is significant. This may be due to multiple factors, including communication challenges, decreased expressive language skills, and limited self-report. Individuals with ID are at a significantly higher risk of having co-morbid medical, genetic, and psychiatric conditions that in turn place them at greater risk for developing medical conditions secondary to substance use.

Patients with ID are less likely to be afforded access to traditional preventative measures and treatment methods. The barriers to treatment have been longstanding and worsen overall health [2]. Individuals with severe and chronic psychiatric illnesses have greatly reduced life expectancy. Co-occurring substance use further reduces their life expectancy. Since many patients with communication deficits exhibit behavioral changes or acute psychiatric symptoms when experiencing medical conditions, the mental health clinician often plays a vital role in facilitating access to appropriate healthcare [3],[4]. Substance use and SUD often goes unrecognized in ID patients. There is a lack of screening and formal assessment for SUD in ID patients. It is important for providers to adequately screen for substance use. Due to a lack of screening and formal assessment of SUD providers may not appreciate the prevalence of substance use among ID patients. This is a missed opportunity for early intervention or prevention and providers may only recognize severe cases.

There is a common misperception that patients with intellectual and/or developmental disabilities do not consume alcohol or use substances. Approximately 5% of patients with ID have a comorbid SUD [5],[6]. These patients frequently abuse alcohol, tobacco, and cannabis, but are largely underdiagnosed and undertreated for SUDs. Treatment for SUDs in these patients is critical because substance abuse among patients with ID is associated with developing mood disorders, long-term health consequences, incarceration, and interpersonal instability.

Individuals with ID suffer disproportionately from substance use problems, due largely to a lack of evidence-supported practices to inform prevention and treatment efforts for them [7]. Past research suggests that prevalence of alcohol and illicit drug use is low in this population, but risk of developing SUD is relatively high among ID substance users due to impulsivity, increased risk of trauma, possible lack of understanding about potential for abuse or consequences of use.

## 2. Substance abuse and mental health services administration

According to Substance Abuse and Mental Health Services Administration (SAMHSA), individuals with traumatic brain injury (TBI), fetal alcohol spectrum disorder, ID, autism spectrum disorder, and other conditions that affect cognition make up a significant proportion of the SUD population [8]. These individuals may have different SUD presentations than those without disabilities. There is limited evidence-based guidance on how to treat these individuals. Mental health providers will benefit from understanding best practices and evidence supported principles for individuals in this population.

Compared with neurotypical patients suffering from SUD, persons with ID who suffer from SUD are less likely to receive treatment or remain in treatment [8],[9]. Research is needed to better gauge the prevalence of substance use problems, identify prevention strategies, and specify treatment components that meet the unique needs of individuals with ID.

## 3. Prevalence

In recent years, rates of lifetime use of both legal and illegal substances has been higher among individuals in the general population as well as those with ID. Current tobacco and alcohol use were shown to be highly prevalent 45.5–48% [9],[10]. Rates for the latter were similar to earlier studies among community samples of individuals with ID. Those individuals with ID living in the least restrictive residential environments were more likely to suffer from SUDs. This patient population is also more likely to experience increased impulsivity, decreased frustration tolerance, and higher rates of both medical and behavioral health conditions, which increases risk and likely leads to higher overall stress levels [9],[10].

## 4. Substance use disorders and treatment

SUD is a serious condition with major consequences, and a variety of potential etiologies. Given the high prevalence of substance use in the ID population, this calls for more attention for identification, prevention, and treatment of SUD [8]. This includes improving access to SUD treatment adapted to the needs of individuals with IDD, improving coping and emotional skills, and promoting a life with adequate social support and community integration.

Motivational interviewing (MI) is a counseling approach developed by Miller and Rollnick (2002) based on the theory that patients enter psychotherapy with varying levels of acceptance of their challenging behaviors and varying amounts of motivation to change them [11]. It is commonly one of the cornerstones of SUD treatment. MI targets the conflicted feelings individuals often experience when thinking about making changes, with the psychotherapist working to help the patient identify, explore, and resolve the person's ambivalence.

To accomplish these objectives, there are four basic techniques that clinicians use in session to help a patient gain insight into the person's problems and to increase motivation to make positive changes [11],[12].

- *Open-ended questions* are used to help facilitate the flow of communication to create forward momentum.
- *Affirmations* are used to help restructure the patient's view of him or herself and their ability to make changes.
- *Reflective listening* is used to help the patient feel that the psychotherapist understands their point of view, as well as to help resolve ambivalence by guiding the patient to explore how the current behavior is impacting their overall quality of life and the benefits of making positive change.
- *Summaries* are a form reflective listening that reviews what has occurred in the session. It is used to draw attention to both sides of the ambivalence the patient is experiencing while promoting the development of discrepancy through careful selection of what information is included or excluded.

## 5. Motivational interviewing adapted for ID

The above MI techniques can be utilized for individuals with ID. Clinicians should take a more directive approach, helping the patient to identify and express feelings regarding the possibility of change. To address any barriers related to communication issues, clinicians should consider utilization of role-playing, visual prompts, pictures, therapeutic games and activities to help facilitate the patient's involvement [11],[12].

## 6. Personality dimensions

Poelen et al. (2017) studied the correlation between personality dimensions and ID/substance use. The aim of this study was to examine the role of the personality dimensions anxiety sensitivity, negative thinking, impulsivity and sensation seeking (as assessed by the revised version of the Substance Use Risk Profile Scale; SURPS) in substance use in individuals with mild to borderline intellectual disabilities (MBID). The authors found no significant relationship between level of ID and the four personality dimensions [12]. In addition, findings showed that individuals with lower levels of anxiety sensitivity, higher levels of negative thinking, impulsivity and sensation seeking showed more severe alcohol use. Individuals with higher levels of negative thinking and sensation seeking had more severe drug use. The SURPS personality dimensions identify persons at increased risk for substance use disorders and might be useful in developing selective substance use interventions for individuals with MBID.

## 7. SAMHSA advisory treatment recommendations

SAMHSA recommends that addiction providers working with people with intellectual disabilities do the following [8]:

- Ask simple questions and repeat them if necessary.
- Teach refusal skills.
- Avoid generalizing, i.e. explain that the same refusal skills that are used at a party can also be used at a bar.
- Have the individual repeat back a concept to make sure they understand.
- Utilize role-playing.
- Having the individual focus on specific goals, i.e. not cashing their SSI check at a liquor store.
- Address trauma in psychotherapy if it is an issue.
- Medication Assisted Treatment.
- Modify Alcoholics Anonymous (AA) groups.

Group therapy can also be effective and some public health entities have successfully adapted the 12-step model to help people with special needs. For example, the Dutchess County Department of Mental Hygiene in New York has provided a modified AA program for people with cognitive disabilities and substance abuse problems since the 1990s.

Existing Program in Sacramento [4],[5],[8]:

- Alta California Research Center-2014 grant.
- Cooperative multi-agency task force.
- Resource manual, modified 12-steps, screening and brief intervention materials, case scenarios.
- Peer mentorship program for individuals with developmental disabilities and substance abuse challenges.
- Community Outreach and Training.
- During the course of the grant 361 community professionals (Kaiser Hospital, Dignity Health, Sacramento County Adult Protective Services) received training and outreach.

Thanks to a research grant Funded by the Mental Health Services Act (MHSA) in partnership with the California Department of Mental Health and Department of Developmental Services Alta California Regional Center (ACRC), a Sacramento-based nonprofit that serves individuals with intellectual developmental, including intellectual, disabilities, obtained a grant which allowed them to help area SUD treatment professionals better identify and work with such individuals [8].

ACRC found that many of the individuals we served were turned down for treatment due to their cognitive delays and the programs expressed they could not adequately meet our client's needs. This collaboration, launched with state grant funds, in part involved:

Developing a brief questionnaire that SUD treatment staff could use to screen clients for developmental disabilities. Convening a joint task force of SUD treatment and developmental disabilities professionals to develop training materials. Cross-training SUD treatment personnel on working with individuals with developmental disabilities [8],[9]. Making SUD treatment staff aware of the support services available through ACRC to clients with developmental disabilities. Cross-training ACRC staff on working with clients with developmental disabilities and SUDs.

A funding opportunity to address a longstanding gap in services through the Mental Health Services Act [8].

The deliverables included:

- Community Steering Committee (MHSA Joint Taskforce).
- Training for alcohol/drug providers.
- Training for Regional Center Service Coordinators.
- Training for Regional Center vendors (developmental disability professionals).
- l Resource manual, modified 12-steps, screening and brief intervention materials, case scenarios.
- Peer mentorship program for individuals with developmental disabilities and substance abuse challenges.
- Community Outreach and Training.
- During the course of the grant 361 community professionals (Kaiser Hospital, Dignity Health, Sacramento County Adult Protective Services) received training and outreach.

## 8. Summary

Be aware that SUDs are common and that all types of substances are used by individuals with ID and careful screening is essential. Prevalence of alcohol and illicit drug use is lower in this population, but risk of developing use disorder is significant. Start early in training refusal skills prior to exposure; social support is critical for reinforcement. Medication Assisted Treatment should be considered in patients with ID just as they are in neurotypical patients. Research to inform prevention and treatment is critical. Well-designed, theory-driven studies of substance use are needed for this population. Priorities to move toward best practices and evidence supported adapted treatment for ID will include research establishing prevalence, research involving efficacy of treatment, and development of standardized screening and assessment instruments of SUD and co-occurring problems targeted to needs of those with ID. In addition, adapted and tailored interventions to the needs of individuals with ID are essential to successful identification and treatment of SUDs. Clearly, research to inform prevention and treatment interventions adapted for ID is much needed.
